# Comparison of oncological outcomes in elderly early-stage cervical cancer patients treated with radical surgery or radiotherapy: A real-world retrospective study with propensity score matching

**DOI:** 10.3389/fonc.2023.1019254

**Published:** 2023-02-15

**Authors:** Yu Gu, Hongyan Cheng, Wei Cang, Lihua Chen, Junjun Yang, Yang Xiang

**Affiliations:** Department of Obstetrics and Gynecology, National Clinical Research Centre for Obstetric and Gynecologic Diseases, Peking Union Medical College Hospital, Chinese Academy of Medical Sciences and Peking Union Medical College, Beijing, China

**Keywords:** cervical cancer, elderly patients, operation, radiotherapy, survival

## Abstract

**Objective:**

To compare the oncological outcomes of radical surgery and radical radiotherapy in elderly (over 65 years) patients with early-stage cervical cancer (IB-IIA).

**Methods:**

Elderly patients with stage IB-IIA cervical cancer treated at Peking Union Medical College Hospital from January 2000 to December 2020 were retrospectively reviewed. All patients were divided into the radiotherapy group (RT group) and the operation group (OP group) according to their primary intervention. Propensity score matching (PSM) analysis was performed to balance the biases. The primary outcome was overall survival (OS), and the secondary outcomes were progression-free survival (PFS) and adverse effects.

**Results:**

A total of 116 patients were eligible for the study (47 in the RT group, and 69 in the OP group), and after PSM, 82 patients were suitable for further analysis (37 in the RT group, and 45 in the OP group). In the real-world setting, it was found that compared with radiotherapy, operation was more frequently selected for elderly cervical cancer patients with adenocarcinoma (P < 0.001) and IB1 stage cancer (P < 0.001). The 5-year PFS rates between the RT and OP groups were not significant (82.3% *vs*. 73.6%, P = 0.659), and the 5-year OS rate of the OP group was significantly better than that in the RT group (100% *vs*. 76.3%, P = 0.039), especially in patients with squamous cell carcinoma (P = 0.029) and tumor size of 2~4 cm with G2 differentiation (P = 0.046). There was no significant difference in PFS between the two groups (P = 0.659). In the multivariate analysis, compared with operation, radical radiotherapy was an independent risk factor of OS (hazard ratio = 4.970, 95% CI, 1.023~24.140, P = 0.047). No difference was observed in adverse effects between the RT and OP groups (P = 0.154) and in ≥grade 3 adverse effects (P = 0.852).

**Conclusion:**

The study found that surgery was more frequently selected for elderly cervical cancer patients with adenocarcinoma and IB1 stage cancer in the real-world setting. After PSM to balance the biases, it showed that compared with radiotherapy, surgery could improve the OS of elderly early-stage cervical cancer patients and was an independent protective factor of OS in elderly early-stage cervical cancer patients.

## Introduction

Cervical cancer currently ranks fourth in female malignancy in terms of incidence and mortality, with 604,127 cases and 341,831 deaths per year (1). Notably, 167,184 elderly women (≥65 years old) are newly diagnosed with cervical cancer every year, accounting for 27.7% of all patients (1, 2). According to the latest American Cancer Society recommendation, individuals aged >65 years with a negative screening history should exit cervical cancer screening (3, 4). Human papilloma virus (HPV) vaccination has been proven to substantially reduce the incidence of cervical cancer and cervical intraepithelial neoplasia in young women (5); however, in China, the age of vaccination is limited to 45 years (6). Therefore, some elderly women over 65 years old seem left to be unvaccinated and unscreened for cervical cancer. Elderly cervical cancer patients often seek medication for obvious symptoms like postmenopausal bleeding. Hence, the screening and treatment of elderly cervical cancer patients are becoming an important public healthcare issue.

Elderly cervical cancer patients are inclined to adopt less aggressive treatment and have a worse prognosis, considering comorbidities and advanced stage (7–9). There is no separate recommendation and discussion for geriatric cervical cancer patients in the latest National Comprehensive Cancer Network guidance (10), and treatment disparities are common in the literature (11–13). Radical surgery is the standard treatment for patients with stages I~IIA, and radiotherapy is recommended for those with stage ≥IIB (10). In clinical practice, radiotherapy is more likely to be recommended to elderly patients with cervical cancer. Given that treatment tolerance is a key concern in geriatric oncology, the choice of intervention in elderly cervical cancer patients remains to be elucidated.

The aim of this respective study was to compare the treatment outcomes of radical surgery and radical radiotherapy in elderly (over 65 years) patients with early-stage cervical cancer (IB-IIA).

## Materials and methods

### Study scheme

All patients with newly diagnosed, biopsy-proved cervical cancer treated at Peking Union Medical College Hospital from January 2000 to December 2020 were identified. The inclusion criteria were 1) aged ≥65 years; 2) squamous carcinoma, adenocarcinoma, and other histopathology like adenosquamous carcinoma; 3) stage IB-IIA (FIGO 2008 and FIGO 2018, according to primary diagnosis time); 4) treated by radical operation or radiotherapy; 5) with complete medical records. Patients were excluded if they 1) were accompanied by other malignancies like ovarian cancer and colorectal cancer or 2) had a follow-up time<3 months. The follow up lasted till December 2021. The study was approved by the institutional review board (S-K 2058).

The demographic characteristics, treatment data, clinicopathological parameters, and follow-up information were collected for each patient. The patients were divided into radiotherapy group (RT group) and operation group (OP group) according to their primary intervention. Radiotherapy included simple radiotherapy and concurrent chemoradiotherapy (CCRT, using cisplatin/paclitaxel); surgery treatment included radical hysterectomy (Querleu–Morrow classification, type C resection) and pelvic/para-aortic lymphadenectomy with/without adjuvant chemotherapy (paclitaxel+cisplatin or paclitaxel+carboplatin), with/without adjuvant radiotherapy. Demographic and clinicopathological characteristics included age, primary complaint, pathology, tumor grade, FIGO stage (all patients were updated to FIGO 2018 stage), tumor size, ThinPrep cytologic test (TCT) result, HPV status, comorbidities, treatment, and adverse effects (according to Common Terminology Criteria for Adverse Events Version 5.0). Follow-up information like the date of last follow-up, the date of recurrence, disease status, site of recurrence, the date of death, and cause of death were obtained *via* telephone calls and outpatient records. The primary outcome was overall survival (OS), and the secondary outcomes were progression-free survival (PFS) and adverse effects. PFS was calculated from the date of treatment to the date of progression, death, or the last follow-up. OS was calculated from the date of treatment to the date of death or the last follow-up. Recurrence was defined as a measurable lesion on imaging (computed tomography, magnetic resonance imaging, or positron emission tomography computed tomography) after the initial radical treatment.

### Statistical analysis

Descriptive statistics and association tests were performed using SPSS Statistics (version 26; IBM Corp., Armonk, NY, USA) and R software (4.1.2). Continuous variables were evaluated with the Mann–Whitney U test (not normal distribution) for group comparison. Categorical variables were evaluated with the Chi-squared test or Fisher’s exact test, and Kruskal–Wallis test for group comparison. Propensity score matching (PSM) analysis was performed to balance the baseline between two groups. Logistic regression was used for the estimation algorithm of PSM, and the matching algorithm used was the nearest neighbor matching. The option for nearest neighbor was set as random matching order, non-replacement, with a caliper of 0.2, and 1:2 matching according to age, FIGO stage, and pathology. Univariate and multivariate Cox proportional hazard analyses were performed to determine risk factors of PFS and OS; the Kaplan–Meier (KM) method was used for survival analysis, and the log-rank test was used for comparisons between groups. All calculated P-values were two-sided, and P-values <0.05 were considered statistically significant.

## Results

**Figure 1:**
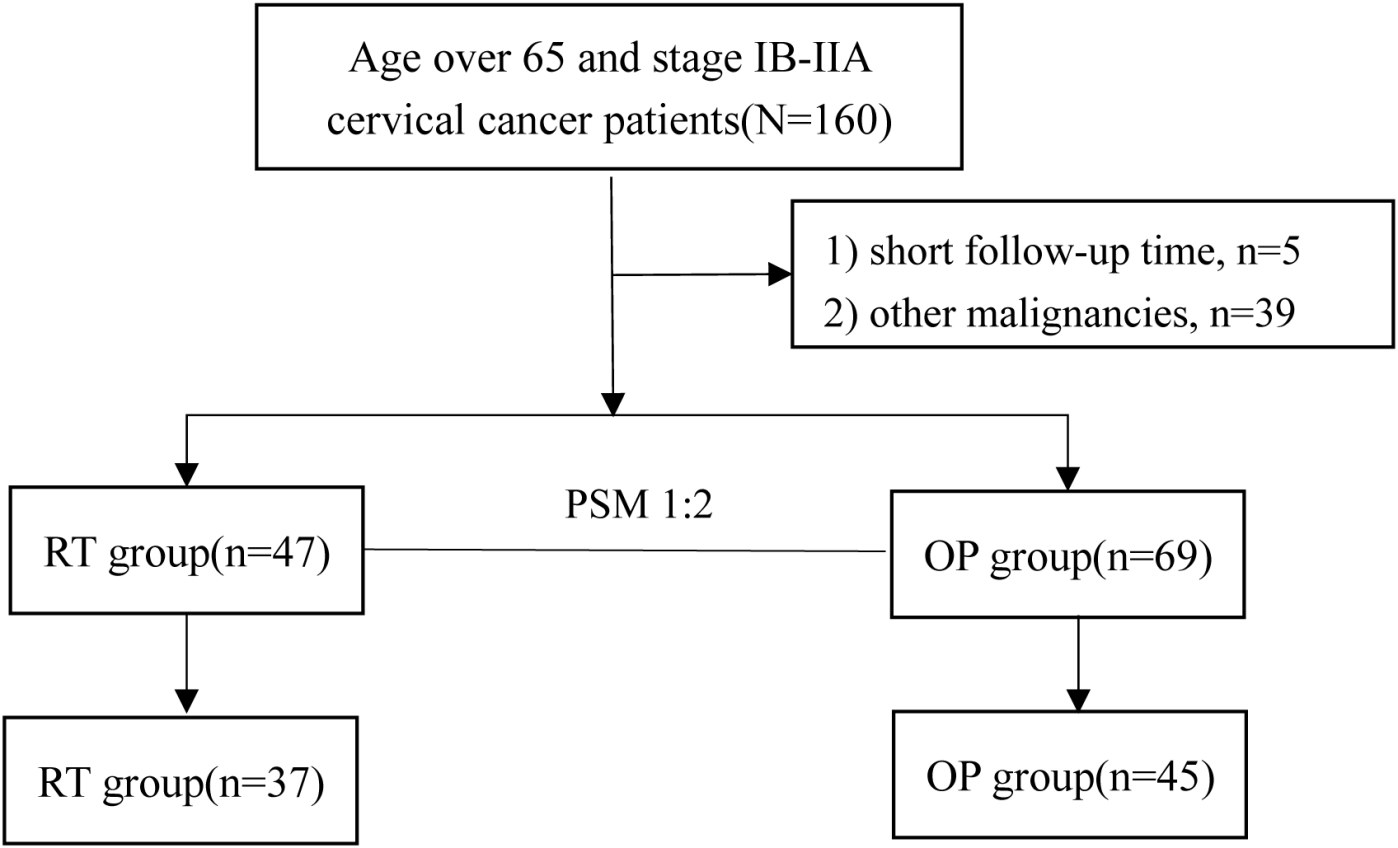
Patient inclusion flow diagram of the study. PSM, propensity score matching; RT, radiotherapy group; OP, operation group.

A total of 116 patients were eligible for the study (47 in the RT group, and 69 in the OP group). The clinical characteristics of the patients are shown in **Table 1**. In the real-world setting, the median age of the entire cohort was 69 (range, 67~72) years and 41.4% (48/116) of patients had comorbidities; 59.5% were initially treated with radical operation, and 8.7% of patients who underwent operation were upgraded to stage IIICp. There were significant differences in age (P = 0.013), pathology (P < 0.001), and FIGO stage (P < 0.001) between the two groups (**Table 1**), indicating that operation was an option for patients who were relatively younger, with adenocarcinoma, and with stage IB1 in clinical practice. After PSM, 82 patients were suitable for further analysis (37 in the RT group, and 45 in the OP group, **Figure 1**), and the baseline characteristics of the two groups were well balanced (**Table 1**).

**Table 1:** Clinical characteristics of patients between two groups before and after PSM.

	Before PSM				After PSM			
	Total	RT	OP	P	Total	RT	OP	P
**Patients (N [%])**	116	47 (40.5)	69 (59.5)		82	37 (45.1)	45 (54.9)	
**Age(median, interquartile range)**	69 (67,72)	71 (67,74)	68 (66,70)	0.013	68.5 (66,72)	69 (66,72.5)	68 (66.5,70.5)	0.506
**Complaint (N [%])**				0.544				0.488
Bleeding	88 (75.9)	38 (80.9)	50 (72.5)		59 (72.0)	28 (75.7)	31 (68.9)	
Abnormal discharge	12 (10.3)	5 (10.6)	7 (10.1)		9 (11.0)	5 (13.5)	4 (8.9)	
Physical examination	15 (12.9)	4 (8.5)	11 (15.9)		13 (15.9)	4 (10.8)	9 (20.0)	
Abdominal discomfort	1 (0.9)	0	1 (1.4)		1 (1.2)	0	1 (2.2)	
**Pathology (N [%])**				<0.001				0.573
Squamous cell carcinoma	83 (71.6)	44 (93.6)	39 (56.5)		73 (89.0)	34 (91.9)	39 (86.7)	
Adenocarcinoma	27 (23.3)	2 (4.3)	25 (36.2)		6 (7.3)	2 (5.4)	4 (8.9)	
Others	6 (5.2)	1 (2.1)	5 (7.2)		3 (3.7)	1 (2.7)	2 (4.4)	
**Grade (N [%])**				0.321				0.152
G1	48 (41.4)	22 (46.8)	26 (37.7)		31 (37.8)	16 (43.2)	15 (33.3)	
G2	53 (45.7)	17 (36.2)	36 (52.2)		38 (46.3)	13 (35.1)	25 (55.6)	
G3	15 (12.9)	8 (17.0)	7 (10.1)		13 (15.9)	8 (21.6)	5 (11.1)	
**FIGO stage (N [%])**				0.002				0.132
IB1	50 (43.1)	15 (31.9)	35 (50.7)		37 (45.1)	14 (37.8)	23 (51.1)	
IB2	16 (13.8)	4 (8.5)	12 (17.4)		11 (13.4)	4 (10.8)	7 (15.6)	
IB3	6 (5.2)	3 (6.4)	3 (4.3)		3 (3.7)	2 (5.4)	1 (2.2)	
IIA1	34 (29.3)	22 (46.8)	12 (17.4)		24 (29.3)	15 (40.5)	9 (20.0)	
IIA2	4 (3.4)	3 (6.4)	1 (1.4)		3 (3.7)	2 (5.4)	1 (2.2)	
IIIC	6 (5.2)	0	6 (8.7)		4 (4.9)	0	4 (8.9)	
**Tumor size (cm)**				0.466				0.719
≤2	67 (57.8)	26 (51.1)	65 (62.3)		50 (61.0)	21 (56.8)	29 (64.4)	
2~4	35 (30.2)	16 (34.0)	19 (27.5)		23 (28.0)	11 (29.7)	12 (26.7)	
>4	14 (12.1)	7 (14.9)	7 (10.1)		9 (11.0)	5 (13.5)	4 (8.9)	
**TCT (N [%])**				0.455				0.553
Normal	66 (56.9)	29 (61.7)	37 (53.6)		45 (54.9)	22 (59.5)	23 (51.1)	
<CIN2	21 (18.1)	6 (12.8)	15 (21.7)		10 (12.2)	3 (8.1)	7 (15.6)	
≥CIN2	29 (25.0)	12 (25.5)	17 (24.6)		27 (32.9)	12 (32.4)	15 (33.3)	
**HPV (N [%])**				0.915				0.744
Normal	79 (68.1)	33 (70.2)	46 (66.7)		54 (65.9)	26 (70.3)	28 (62.2)	
16 ± 18 positive	19 (16.4)	7 (14.9)	12 (17.4)		15 (18.3)	6 (16.2)	9 (20.0)	
Others positive	18 (15.5)	7 (14.9)	11 (15.9)		13 (15.9)	5 (13.5)	8 (17.8)	
**Comorbidities (N [%])**								
Diabetes	21 (18.1)	10 (21.3)	11 (15.9)	0.464	16 (19.5)	8 (21.6)	8 (17.8)	0.781
Hypertension	42 (36.2)	14 (29.8)	28 (40.6)	0.235	30 (36.6)	10 (27.0)	20 (44.4)	0.114
Cardiovascular disease	10 (8.6)	4 (8.5)	6 (8.7)	0.972	8 (9.8)	2 (5.4)	6 (13.3)	0.284

PSM, propensity score matching; RT, radio-chemotherapy group; OP, operation group; TCT, ThinPrep cytologic test; CIN, cervical intraepithelial neoplasia; HPV, human papilloma virus.

**Figure 2:**
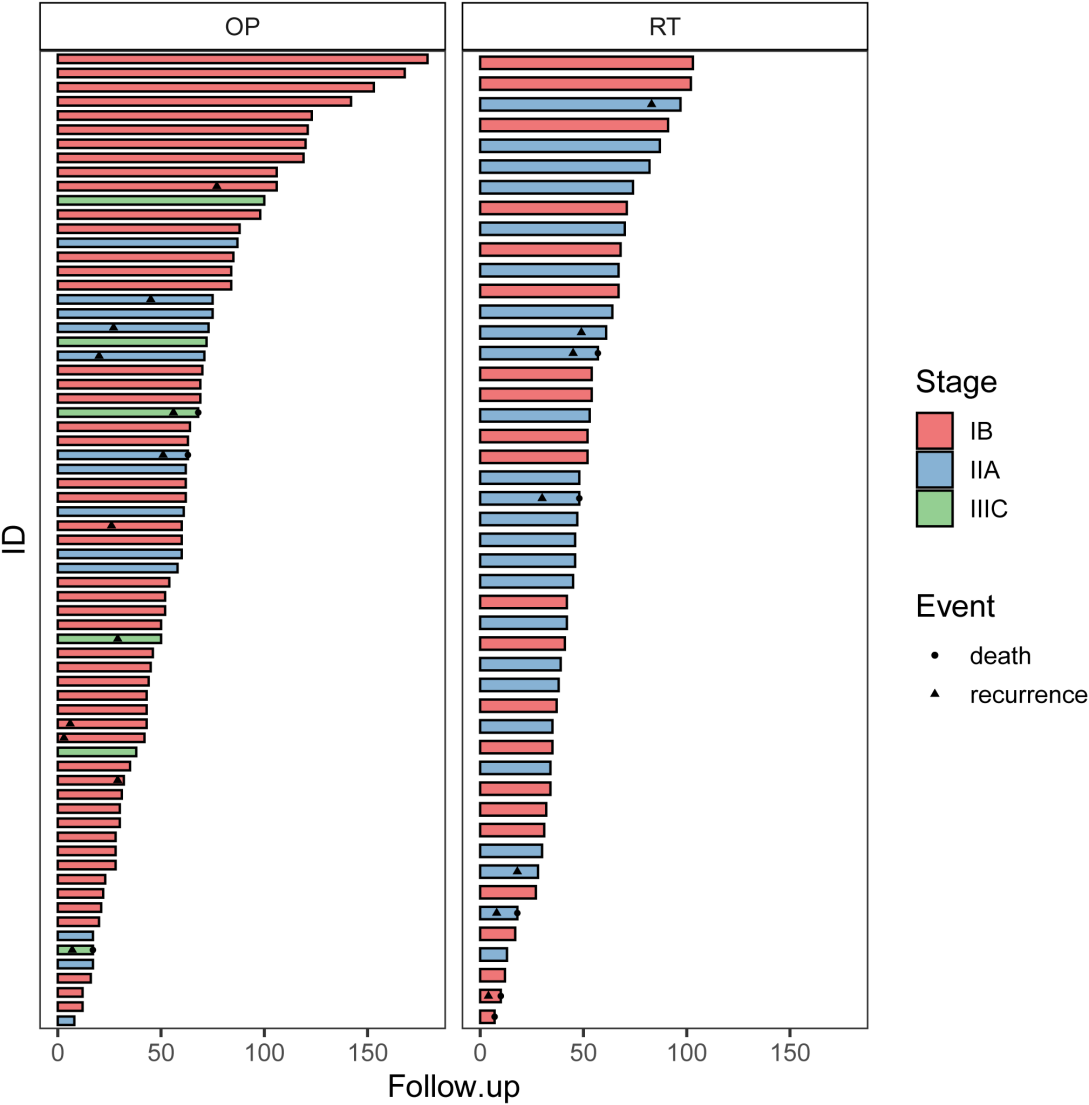
The swimmer plots of patients in the OP and RT groups. OP, operation group; RT, radiotherapy group.

**Figure 3:**
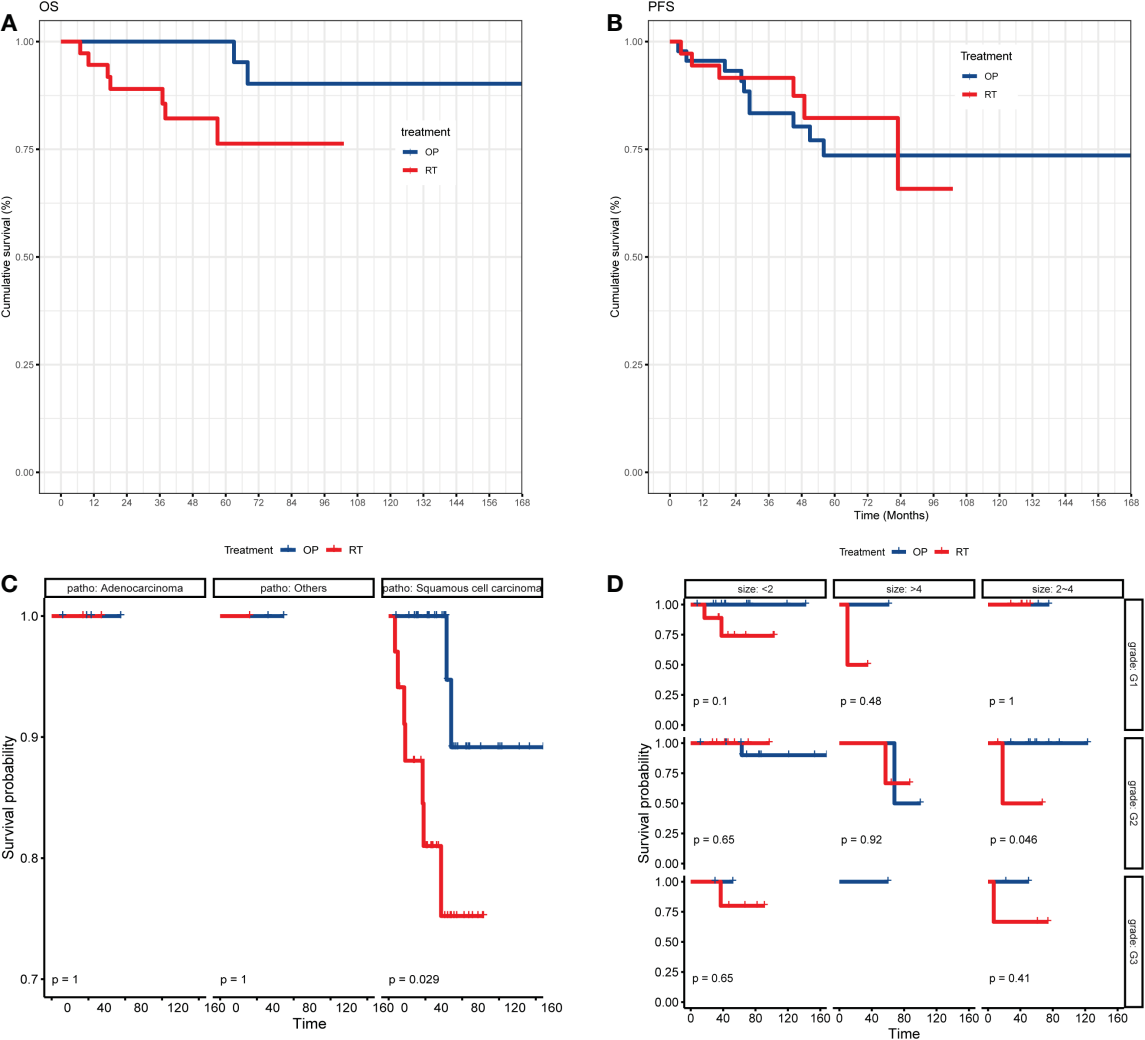
The KM analysis of OS and PFS of patients between the OP and RT groups. **(A)** KM analysis of OS between the OP and RT groups; **(B)** KM analysis of PFS between the OP and RT groups; **(C)** subgroup analysis of OS between the OP and RT groups stratified by pathology; **(D)** subgroup analysis of OS between the OP and RT groups stratified by grade and tumor size. OP, operation group; RT, radiotherapy group.

The clinical courses of each patient in the two groups are demonstrated in the swimmer plots (**Figure 2**). With a median follow-up of 55.5 (range, 7~168) months in the matched cohort, the median PFS of the RT and OP groups was 47 (range, 0~103) months and 52 (range, 3~168) months, respectively, and the median OS was 47 (range, 7~103) months and 62 (range, 3~168) months, respectively. The 5-year PFS rates in the RT and OP groups were 82.3% (95% confidence interval [CI], 61.2%~92.5%) and 73.6% (95% CI, 55.9%~85.1%), respectively, and the 5-year OS rates were 76.3% (95% CI, 54.9%~88.5%) and 100% (95% CI, NA), respectively. In the KM analysis, the OP group showed better OS than the RT group (log rank, 5.280, P = 0.039; **Figure 3A**), especially in patients with squamous cell carcinoma (P = 0.029) and a tumor size of 2~4 cm with G2 differentiation (P = 0.046), according to the subgroup analysis stratified by pathology, tumor size, stage, and grade (**Figures 3C-D**, **Figures S1.1-S1.2**). There was no significant difference in PFS between the two groups (log-rank, 0.80, P = 0.659; **Figure 3B**), and no differences were recognized in the subgroup analysis (P > 0.05, **Figures S1.3-S1.4**).

In the multivariate analysis, compared with operation, radical radiotherapy was an independent risk factor of OS (hazard ratio, [HR] = 4.970, 95% CI, 1.023~24.140, P = 0.047; **Table 2**). Treatment was not an independent prognostic factor for PFS (P = 0.659), and no other independent risk factors were identified in the multivariate analysis of PFS (**Table 3**). Furthermore, univariate and multivariate analyses of PFS and OS in patients with squamous cell carcinoma were performed and no risk factors were identified (**Tables S1-S2**).

**Table 2:** Univariate and multivariate analyses of OS in the post-PSM cohort (N = 82).

			Univariate			Multivariate		
		N	P	HR	95% CI	P	HR	95% CI
Stage								
	>IB	31	Ref					
	IB	51	0.378	0.553	0.148~2.063			
Pathology								
	Non-SCC	9	Ref					
	SCC	73	0.99840	76977448	0~Inf			
Tumor size (cm)								
	≤2	50	Ref			Ref		
	2~4	23	0.787	1.265	0.230~6.949	0.773	1.285	0.233~7.092
	>4	9	0.067	4.079	0.909~18.314	0.092	3.658	0.811~16.500
Grade								
	G1	31	Ref					
	G2	38	0.867	0.879	0.195~3.956			
	G3	13	0.621	1.571	0.262~9.416			
Treatment								
	OP	45	Ref			Ref		
	RT	37	0.0387	5.279	1.091~25.550	0.047	4.97	1.023~24.140

SCC, squamous cell carcinoma; OP, operation group; RT, radio-chemotherapy group.

**Table 3:** Univariate and multivariate analyses of PFS in the post-PSM cohort (N = 82).

			Univariate			Multivariate		
		N	P	HR	95% CI	P	HR	95% CI
Stage								
	>IB	31	Ref			Ref		
	IB	51	0.016	0.272	0.094~0.786	0.056	0.323	0.103~1.027
Pathology								
	Non-SCC	9	Ref					
	SCC	73	0.997	78616445	0~Inf			
Tumor size (cm)								
	≤2	50	Ref			Ref		
	2~4		0.333	1.767	0.558~5.597	0.604	1.367	0.419~4.459
	>4		0.064	3.199	0.935~10.938	0.415	1.743	0.458~6.630
Grade								
	G1	31	Ref					
	G2	38	0.746	0.839	0.289~2.434			
	G3	13	0.736	0.759	0.153~3.766			
Treatment								
	OP	45	Ref					
	RT	37	0.659	0.795	0.288~2.197			

OP, operation group; RT, radio-chemotherapy group.

At the last follow-up, 16 patients relapsed (six in the RT group and 10 in the OP group) and nine patients died (seven in the RT group and two in the OP group). The common recurrence sites were the vaginal stump (8/16), pelvic mass (1/16), lung (1/16), liver (1/16), and bone (1/16). In the OP group, 39.1% of patients underwent operation by the transabdominal approach and 56.5% using the laparoscopic approach before PSM; after PSM, the proportions were 33.3% and 60%, respectively. There were no significant differences in PFS and OS between patients who underwent the laparoscopic and transabdominal approaches before and after PSM (P > 0.05, **Figures S2A-D**). Moreover, in the real-world setting, 82.6% (57/69) of patients endured adjuvant therapy after operation (46 with chemoradiotherapy, and 11 with chemotherapy), and after PSM, 73.3% (33/45) of patients endured adjuvant therapy after operation (32 with chemoradiotherapy, and one with chemotherapy). As for adverse effects, there were 18 cases in the RT group, mainly including eight cases of myelosuppression (four patients ≥grade 3), five cases of radiation enteritis (one patients ≥grade 3), and four cases of urinary tract infection (one patient ≥grade 3). There were 30 cases in the OP group, mainly including seven cases of urinary tract infection (three patients ≥grade 3), seven cases of myelosuppression (three patients ≥grade 3), three cases of lymphedema, and three cases of radiation enteritis. No difference was observed in adverse effects between the RT and OP groups (18 *vs*. 30, χ2 = 2.028, P = 0.154), and there was no difference in ≥grade 3 adverse effects between the two groups (six *vs*. eight, χ2 = 0.035, P = 0.852).

## Discussion

The current study found that operation was an option for elderly patients who were relatively younger (P = 0.013), with adenocarcinoma (P < 0.001), and with stage IB1 (P < 0.001) in real-world clinical practice. After PSM, the baseline characteristics of the two groups were well balanced. The 5-year PFS rates in the RT and OP groups were 82.3% (95% CI, 61.2%~92.5%) and 73.6% (95% CI, 55.9%~85.1%), and the 5-year OS rates were 76.3% (95% CI, 54.9%~88.5%) and 100% (95% CI, NA). The OP group showed better OS than the RT group (P = 0.039). In the multivariate analysis, compared with operation, radiotherapy was an independent risk factor for OS (HR = 4.970, P = 0.047). No difference was observed in adverse effects between the RT and OP groups (χ2 = 2.028, P = 0.154).

Cervical cancer occurs at two age peaks, one at 40 years of age and the other at 60~70 years of age (14). Recently, elderly cervical cancer patients have received increased attention, but the definition of “elderly” is heterogeneous, including those aged over 70, 65, or 60 years (8, 15–18). Approximately 20%–27% of cervical cancer patients are aged over 65 years; hence, the current study selected the cohort of patients aged >65. Elderly patients tend to have more comorbidities, such as diabetes and cardiovascular disease, which influence the choice of treatment and deteriorate elderly patient survival (8, 11, 13, 19, 20). Eggemann et al. (21) found that elderly patients comprised 35% of all cervical cancer patients, and patients older than 60 years were more likely to have undifferentiated tumors and less likely to receive operation (odds ratio 0.39; 95% CI 0.20–0.77). A study based on the Surveillance, Epidemiology, and End Results Program database showed that compared with <65-year-old patients, elderly cervical cancer patients had more advanced disease, few of whom chose operation, and had significantly worse 5-year OS (59.38% *vs*. 75.02%, P < 0.001) (15). Therefore, the diagnosis of cervical cancer is often delayed and undertreated in elderly cervical cancer patients, compared with younger patients, which may be correlated with poor prognosis.

In cervical cancer patients with early-stage cancer, operation is considered as the standard treatment (22); however, in elderly patients, the feasibility of surgery is a major concern. In this real-world study, it was found that compared with radiotherapy, operation was more frequently selected for elderly cervical cancer patients with adenocarcinoma and IB1 stage cancer, and 59.5% of patients underwent radical operation. Similarly, in a cohort of 64 senior cervical cancer patients with stage I–IV cancer reported by Barben et al. (23), the operation rate in early-stage patients was 45.5%. In another study of 2,247 cervical cancer patients over 65 years of age, 54.3% received operation (24). Xie et al. (15) also focused on the clinical characteristics of cervical cancer over 65 years of age, and they found that 62.3% of the patients were initially treated using surgery. Hence, in clinical practice, it seems that operation is feasible in over half of the elderly cervical cancer patients.

After PSM to balance the aforementioned bias, it was shown that operation could improve the OS of elderly early-stage cervical cancer patients, and operation was an independent protective factor of OS in elderly early-stage cervical cancer patients. Similarly, a study by Xie et al. (15) also supported that operation could improve cancer-specific survival in patients aged over 65 years. The choice of operation should be personalized, rather than one-fits-all. Although there were no differences in PFS and OS of patients with laparoscopic and transabdominal approaches before and after PSM in this study, numerous retrospective studies have demonstrated the feasibility of operation, even the benefit of minimal invasive surgery, in elderly cervical cancer patients (25–28). In comparison, two studies (29, 30) have questioned the efficacy of a minimal access approach for cervical cancer since 2018, and the open abdominal approach has been recommended as the standard approach for early-stage cervical cancer patients (31, 32). The choice of surgery and its approach in elderly cervical cancer patients should be determined by the clinician and based on operation risk stratification tools for elderly patients (33, 34). Further studies were needed to provide more evidence.

Considering the low feasibility of surgery in some elderly patients, radiation therapy is a commonly used curative option. Previous studies have confirmed the efficiency and safety of radiation therapy in elderly patients with cervical cancer (12, 35, 36). Recently, remarkable progress has been made in radical radiotherapy for cervical cancer treatment. It is widely considered that cervical cancer patients, including elderly patients, can benefit from concurrent chemoradiotherapy (CCRT) compared with radical radiotherapy (12, 37, 38). Additionally, it is striking that sequential chemoradiation, rather than CCRT, can improve oncological outcomes in women with early-stage cervical cancer after radical operation (39, 40). Further investigations are warranted to determine its efficacy in elderly patients.

This study has some limitations. Firstly, this study was retrospective in nature and had intrinsic disadvantages. Secondly, this was a real-world study and the imbalance between groups regarding age and stage reflects the realistic clinical choice for patients, which may introduce bias for conclusion. Therefore, further prospective studies with large sample sizes are needed.

## Conclusion

The study found that surgery was more frequently selected for elderly cervical cancer patients with adenocarcinoma and IB1 stage cancer in the real-world setting. After PSM to balance the biases, it showed that compared with radiotherapy, surgery could improve the OS of elderly early-stage cervical cancer patients and was an independent protective factor of OS in elderly (age >65 years) early-stage (IB~IIA) cervical cancer patients.

## Data availability statement

The original contributions presented in the study are included in the article/**Supplementary Material**. Further inquiries can be directed to the corresponding author.

## Ethics statement

Written informed consent was obtained from the individual(s) for the publication of any potentially identifiable images or data included in this article.

## Author contributions

YG and HC collected data and drafted the manuscript. WC and LC performed statistical analysis and data interpretation. JY revised the manuscript. YX designed the study. All authors contributed to the article and approved the submitted version.

## Supplementary material

Supplementary Figure 1 The subgroup analysis of OS and PFS of patients between the OP and RT group. 1.1A~1.1C) the KM analysis of OS between the OP and RT group stratified by stage(1.1A), tumor size(1.1B), grade(1.1C); 1.2A~1.2D) the subgroup analysis of OS between the OP and RT group stratified by stage and pathology(1.2A), stage and grade(1.2B), pathology and tumor size(1.2C), pathology and grade(1.2D); 1.3A~1.3D) the KM analysis of PFS between the OP and RT group stratified by stage(1.3A), tumor size(1.3B), pathology(1.3A) and grade(1.3D); 1.4A~1.4E) the subgroup analysis of OS between the OP and RT group stratified by stage and pathology(1.4A), stage and grade(1.4B), pathology and tumor size(1.4C), grade and tumor size(1.4D), pathology and grade(1.4E). OP: operation group, RT: radiotherapy group.

Supplementary Figure 2 The KM analysis of PFS and OS of patients in the OP group stratified by operation approach before and after PSM. S2A) the KM analysis of PFS between patients with laparoscopic and transabdominal approach before PSM; S2B) the KM analysis of PFS between patients with laparoscopic and transabdominal approach after PSM; S2C) the KM analysis of OS between patients with laparoscopic and transabdominal approach before PSM; S2D) the KM analysis of OS between patients with laparoscopic and transabdominal approach after PSM; OP: operation group, PSM: propensity score matching.
