# Supplementary figures and images for: Comparison of oncological outcomes in elderly early-stage cervical cancer patients treated with radical surgery or radiotherapy: A real-world retrospective study with propensity score matching
Source: Front Oncol. 2023 Feb 15;13:1019254. doi: 10.3389/fonc.2023.1019254 (PMC9975559; doi:10.3389/fonc.2023.1019254)

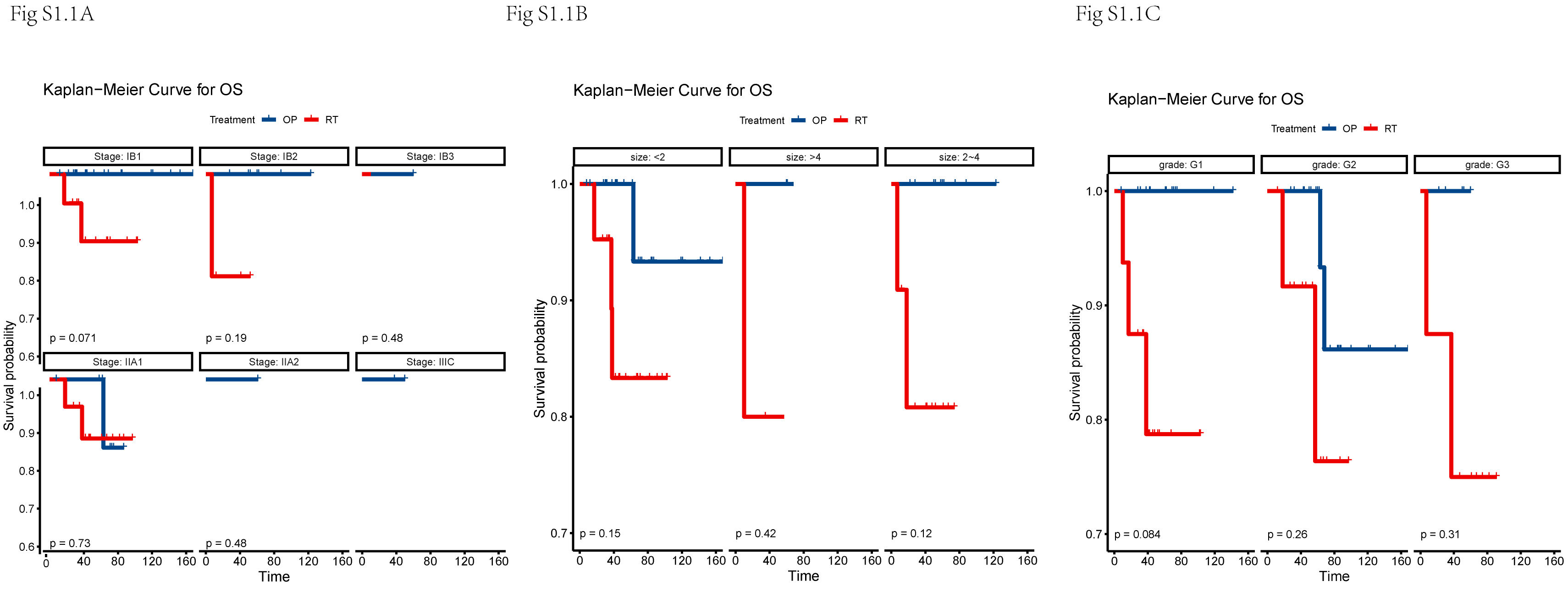

Supplement: Supplementary Figure 1 — The subgroup analysis of OS and PFS of patients between the OP and RT group. 1.1A~1.1C) the KM analysis of OS between the OP and RT group stratified by stage(1.1A), tumor size(1.1B), grade(1.1C); 1.2A~1.2D) the subgroup analysis of OS between the OP and RT group stratified by stage and pathology(1.2A), stage and grade(1.2B), pathology and tumor size(1.2C), pathology and grade(1.2D); 1.3A~1.3D) the KM analysis of PFS between the OP and RT group stratified by stage(1.3A), tumor size(1.3B), pathology(1.3A) and grade(1.3D); 1.4A~1.4E) the subgroup analysis of OS between the OP and RT group stratified by stage and pathology(1.4A), stage and grade(1.4B), pathology and tumor size(1.4C), grade and tumor size(1.4D), pathology and grade(1.4E). OP: operation group, RT: radiotherapy group. [file Image_1.tif]

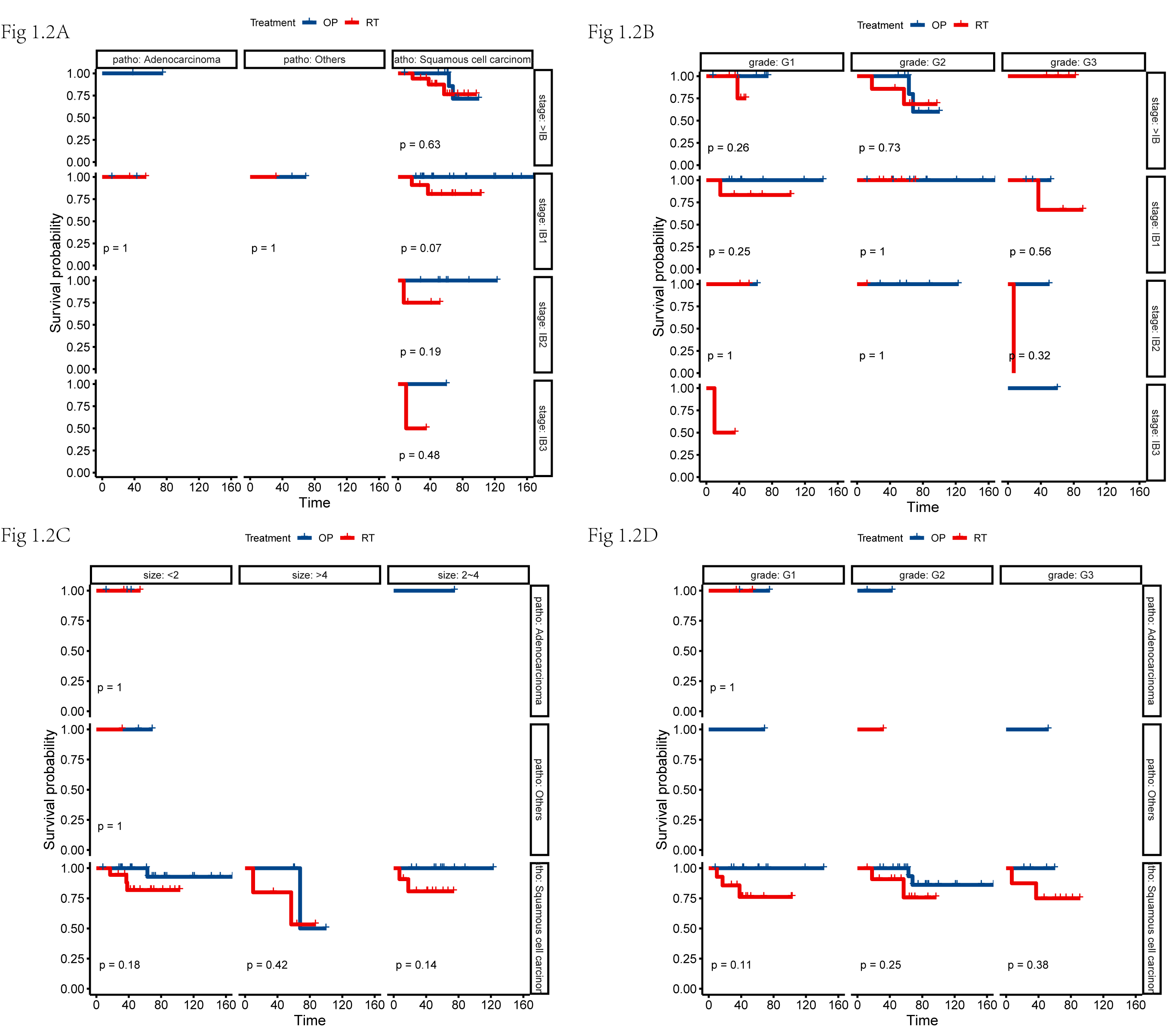

Supplement: Supplementary Figure 2 — The KM analysis of PFS and OS of patients in the OP group stratified by operation approach before and after PSM. S2A) the KM analysis of PFS between patients with laparoscopic and transabdominal approach before PSM; S2B) the KM analysis of PFS between patients with laparoscopic and transabdominal approach after PSM; S2C) the KM analysis of OS between patients with laparoscopic and transabdominal approach before PSM; S2D) the KM analysis of OS between patients with laparoscopic and transabdominal approach after PSM; OP: operation group, PSM: propensity score matching. [file Image_2.tif]

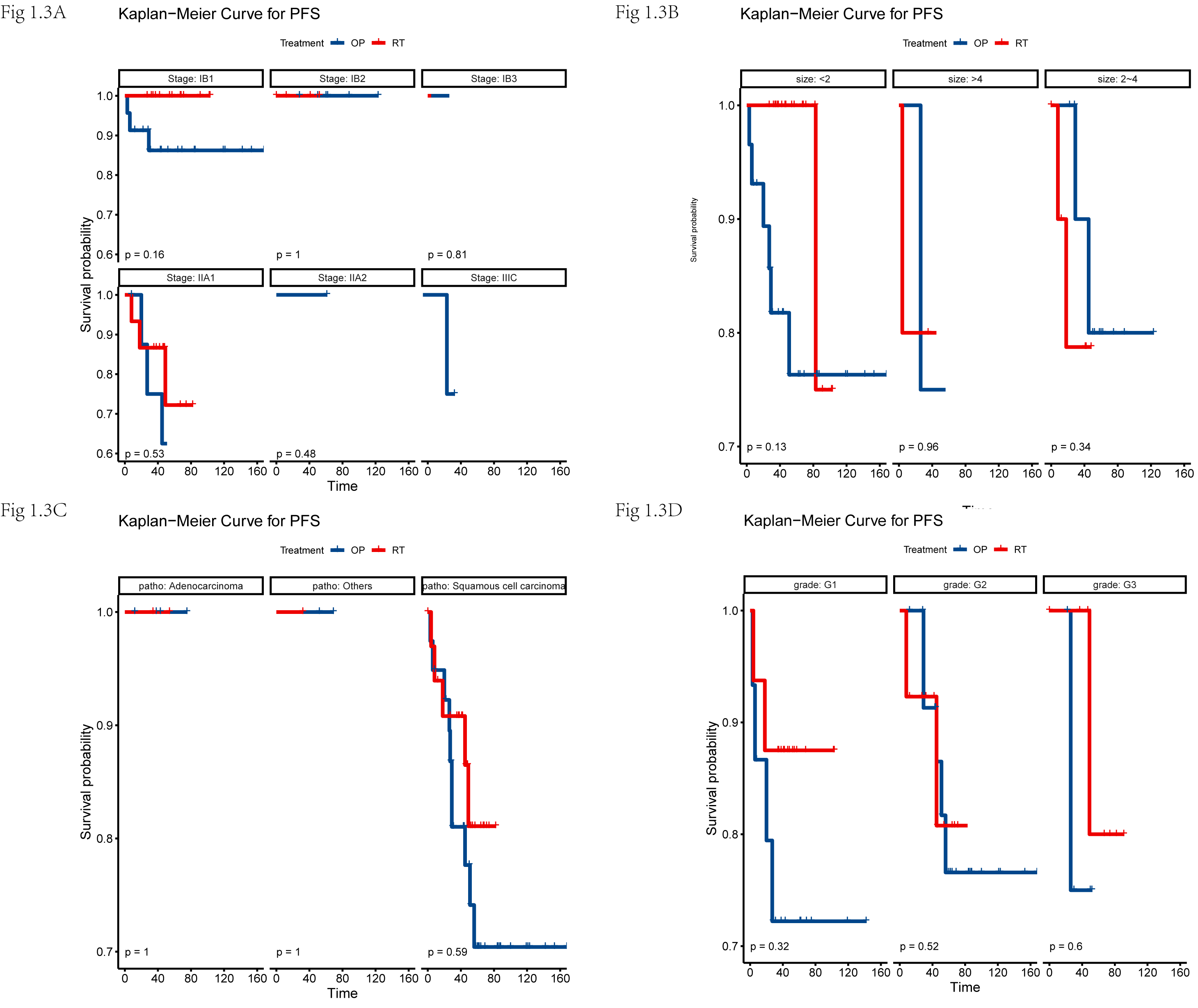

Supplement: Supplementary file 3 [file Image_3.tif]

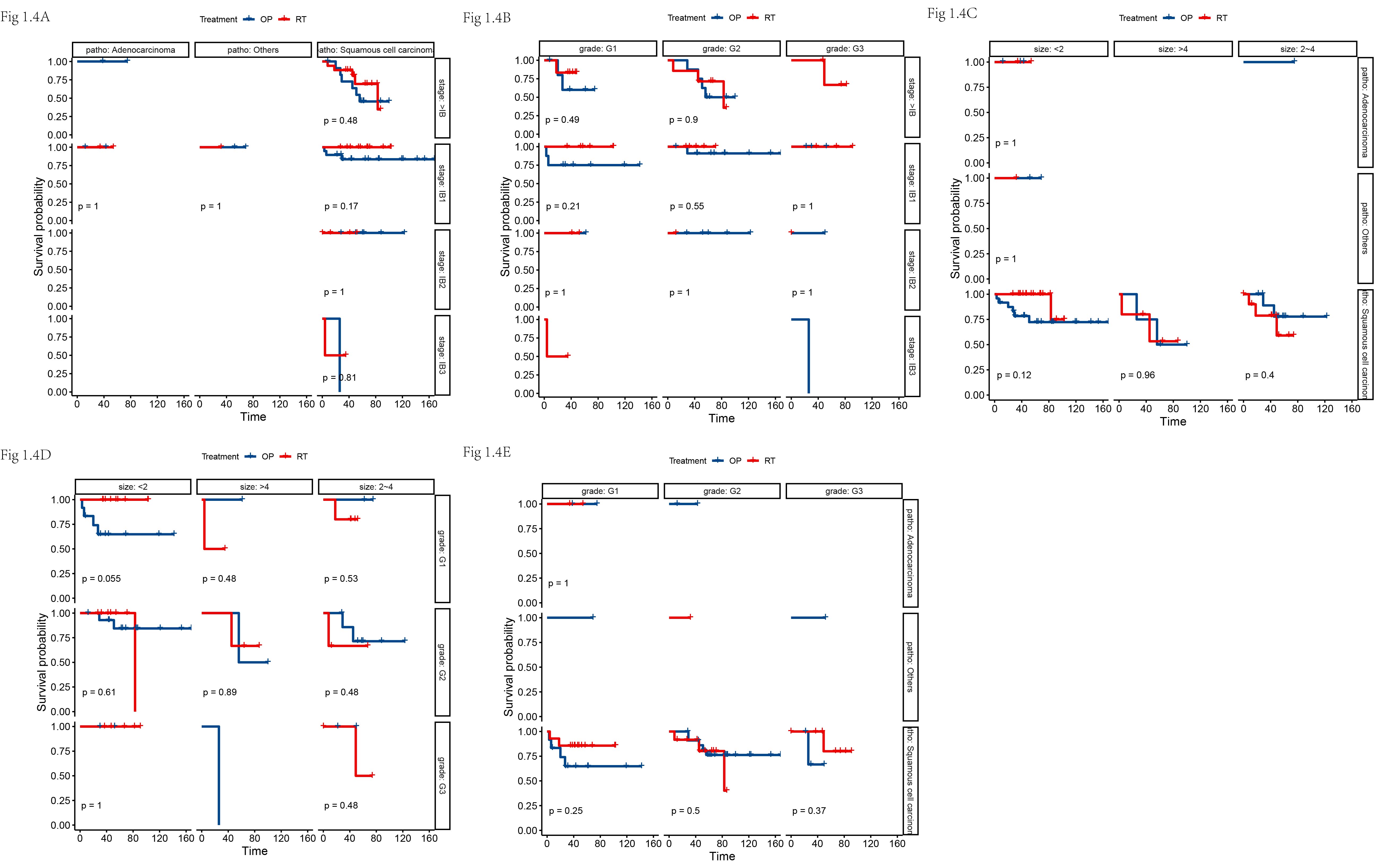

Supplement: Supplementary file 4 [file Image_4.jpeg]

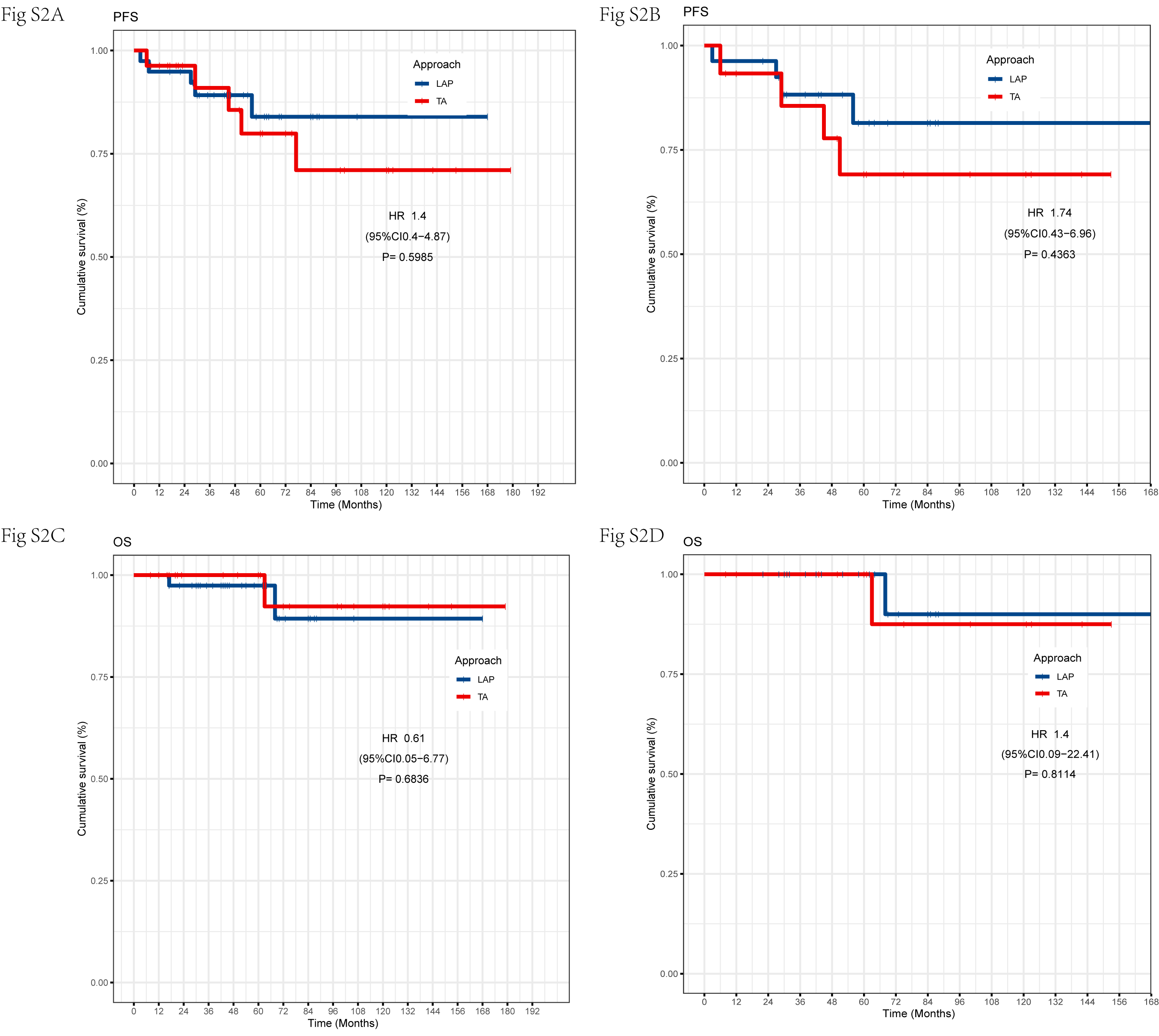

Supplement: Supplementary file 5 [file Image_5.tif]
