# Supplementary material for: Comparison of oncological outcomes in elderly early-stage cervical cancer patients treated with radical surgery or radiotherapy: A real-world retrospective study with propensity score matching
Source: Front Oncol. 2023 Feb 15;13:1019254. doi: 10.3389/fonc.2023.1019254 (PMC9975559; doi:10.3389/fonc.2023.1019254)
Supplement: Supplementary file 6 [file Table_1.docx]

**Table S1 Univariate and multivariate analysis of OS in patients with Squamous cell carcinoma from post PSM cohort(N=73).**

|  |  |  | **Univariate** | | | **Multivariate** | | |
| --- | --- | --- | --- | --- | --- | --- | --- | --- |
|  |  |  | P | HR | 95%CI | P | HR | 95%CI |
| Stage |  |  |  |  |  |  |  |  |
|  | >IB | 29 | ref |  |  |  |  |  |
|  | IB | 44 | 0.407 | 0.573 | 0.154~2.138 |  |  |  |
| Tumor size(cm) |  |  |  |  |  |  |  |  |
|  | ≤2 | 42 | ref |  |  | ref |  |  |
|  | 2~4 | 22 | 0.825 | 1.213 | 0.219~6.704 | 0.835 | 1.199 | 0.216~6.652 |
|  | >4 | 9 | 0.093 | 3.625 | 0.808~16.27 | 0.117 | 3.333 | 0.7397~15.02 |
| Grade |  |  |  |  |  |  |  |  |
|  | G1 | 26 | ref |  |  |  |  |  |
|  | G2 | 35 | 0.703 | 0.746 | 0.165~3.367 |  |  |  |
|  | G3 | 12 | 0.73 | 1.371 | 0.229~8.218 |  |  |  |
| Treatment |  |  |  |  |  |  |  |  |
|  | OP | 39 | ref |  |  | ref |  |  |
|  | RT | 34 | 0.049 | 4.887 | 1.011~23.632 | 0.055 | 4.672 | 0.9641~22.64 |

**Table S2 Univariate and multivariate analysis of PFS in patients with Squamous cell carcinoma from post PSM cohort(N=73).**

|  |  |  | **Univariate** | | | **Multivariate** | | |
| --- | --- | --- | --- | --- | --- | --- | --- | --- |
|  |  | N | P | HR | 95%CI | P | HR | 95%CI |
| Stage |  |  |  |  |  |  |  |  |
|  | >IB | 29 | ref |  |  |  |  |  |
|  | IB | 44 | 0.022 | 0.289 | 0.1~0.836 | 0.058 | 0.331 | 0.1059~1.036 |
| Tumor size(cm) |  |  |  |  |  |  |  |  |
|  | ≤2 | 42 | ref |  |  |  |  |  |
|  | 2~4 | 22 | 0.406 | 1.632 | 0.514~5.175 | 0.61 | 1.356 | 0.4206~4.369 |
|  | >4 | 9 | 0.103 | 2.783 | 0.814~9.518 | 0.499 | 1.579 | 0.4207~5.923 |
| Grade |  |  |  |  |  |  |  |  |
|  | G1 | 26 | ref |  |  |  |  |  |
|  | G2 | 35 | 0.528 | 0.709 | 0.244~2.061 |  |  |  |
|  | G3 | 12 | 0.636 | 0.679 | 0.137~3.371 |  |  |  |
| Treatment |  |  |  |  |  |  |  |  |
|  | OP | 39 | ref |  |  |  |  |  |
|  | RT | 34 | 0.585 | 0.754 | 0.273~2.082 |  |  |  |
